# Employment of a needs assessment survey to shape a novel web-based pediatric rheumatology curriculum for primary care providers

**DOI:** 10.1186/1546-0096-11-26

**Published:** 2013-06-05

**Authors:** Amy Louise Woodward, Zena Leah Harris

**Affiliations:** 1 Department of Pediatrics, Vanderbilt University School of Medicine, Nashville, TN, USA; 2 Department of Pediatrics, Northwestern University Feinberg School of Medicine, Chicago, IL, USA

## Abstract

**Background:**

Pediatric rheumatology faces many challenges due to the shortage of board certified physicians in the field and the imbalance in their geographic distribution. This shortage has required primary care physicians and adult rheumatologists to assume the care of children with rheumatologic diseases, though these physicians report significant discomfort doing so. We are addressing this issue through the development of a novel web-based curriculum aimed at primary care physicians.

**Methods:**

We pursued a needs assessment survey of Vanderbilt pediatric residency graduates (1981–2010) working in primary care. Our goals were to understand their perceptions of what the needs are and what educational interventions would be most effective.

**Results:**

Of 152 surveys sent successfully via Survey Monkey, we received 28 responses (18.4%). Responses suggest there to be a discrepancy between physicians’ general assessment of their training and their self-reported ability to recognize specific diseases. Nearly 80% of respondents felt that additional education in pediatric rheumatology would improve their ability to co-manage children with the rheumatologist. Action plans for common rheumatologic complaints and potential emergencies were thought to be of potential benefit by a majority of respondents.

**Conclusions:**

We will utilize our survey results to develop a learner centered curriculum to have the highest positive impact in assisting primary care providers in caring for children with rheumatologic diseases.

## Background

The care for children with rheumatologic diseases faces major challenges, many of which are tied to the long-recognized shortage of board certified pediatric rheumatologists in the U.S. An American College of Rheumatology (ACR) workforce study projects that even though the number of pediatric rheumatologists is rising, the increase in demand will outpace these gains over the next 20 years [1]. In addition, there is a gross imbalance of geographic distribution; 40% of U.S. children live more than 40 miles from a pediatric rheumatologist; 24% live more than 80 miles from such care [2]. Adult rheumatologists and primary care physicians have therefore had to assume the care of these children, even though these physicians report inadequate training and significant discomfort in doing so [3-6]. This unease is not surprising given that one third of U.S. medical schools are without a pediatric rheumatologist, as are more than 40% of pediatric residency programs [7]. While lack of exposure to rheumatology during pediatric residency hurts recruitment into the field, on a more basic level, graduates enter practice unprepared to recognize autoimmune diseases.

While there are significant efforts underway nationally to increase the number of pediatric rheumatologists, from a practical standpoint, it will take several years for new trainees to enter the workforce. We have therefore embarked on a project to develop a novel web-based interactive educational resource linking off the Vanderbilt Department of Pediatrics website. This resource will be aimed at primary care providers, but also will have application for pediatric residents still in training. Our specific goals with this project are: 1) To avoid delayed diagnoses of treatable autoimmune diseases in children, thereby minimizing the probability of poor outcomes such as irreversible joint damage; 2) To increase the effectiveness of collaborative care between primary and subspecialty care physicians when access to a pediatric rheumatologist is limited; 3) To help attract residents to careers in pediatric rheumatology.

For our website to have the highest positive impact, we sought to understand the perceptions of primary care physicians regarding what the needs are, and what educational interventions would be most effective. We therefore developed a needs assessment survey and distributed this to graduates of the Vanderbilt pediatric residency program. We partnered with the Vanderbilt Medical Alumni Association (VMAA) to accomplish this, and were given permission to use their large database for our survey.

## Methods

After receiving approval of the Vanderbilt Institutional Review Board (IRB), the VMAA supplied us with a list of graduates from the years 1981–2010. We subsequently narrowed the list to those graduates who went into primary care rather than the entire graduate roster. We felt that targeting those graduates working in primary care would increase the relevance of our survey, as primary care physicians are our primary audience for educational interventions. We further narrowed the list to those who both graduated from either a pediatric or medicine-pediatric residency training program and maintained active contact information including e-mail addresses with the VMAA office. We utilized e-mail (Survey Monkey) rather than envelope mailings, which was both cost-saving from a budget standpoint, as well as advantageous for subsequent data analysis. To increase participation, the Associate Dean for Alumni Affairs included an introductory message stating her support for this project. The survey also linked back to the VMAA website on completion of the questions. The survey was distributed in December 2010, with a follow-up distribution in January 2011. The survey was closed at the end of January 2011.

A total of 178 surveys were distributed, with 26 surveys bouncing back due to an invalid e-mail address. This left 152 surveys sent successfully. We had a total of 28 responses at the close of the survey, or a response rate of 18.4%.

The survey itself was not in the format of a knowledge assessment or quiz for the respondent. Questions instead probed what the physicians felt they did or did not learn during residency, and asked directly how we might improve collaborative care with the rheumatologist. The survey was structured into 4 major sections:

I) Demographics: Though the survey was anonymous, we asked general demographic questions including zip code of their practice location and distance from a pediatric rheumatologist.

II) General knoweldge base: Assessment of their general knowledge base gained during residency regarding autoimmune diseases and musculoskeletal disorders. This section included a series of questions regarding their comfort level at the end of residency with the basic joint exam, basic inflammatory labs, autoimmune serologies, and the initial work-up of presenting symptoms such as musculoskeletal pain and muscle weakness.

III) Knowledge of specific rheumatologic diseases: Assessment of their ability on completion of residency to recognize specific autoimmune diseases such as chronic arthritis, dermatomyositis and lupus.

IV) Ability to co-manage patients with their rheumatologist: Questions regarding their comfort level co-managing patients with a rheumatologist, including means by which their ability to co-manage these patients might be improved.

## Results

### Section I: demographics

Answers, while de-identified, allowed for linkage by practice zip code and year that they completed their residency training. Of the 28 respondents, 2 were no longer practicing. Of the remaining 26, the majority practice in Tennessee (16/26 ~ 62%) with a remaining geographic spread extending from Massachusetts to Colorado. Only 7/26 practices were within a 30 minute or less car drive to a pediatric rheumatologist, with the median distance being 50 miles. The most extreme distance a practice was from a pediatric rheumatologist was 200 miles.

The 28 respondents completed residency between 1984 and 2009. We went on to inquire if they had received any sub-specialty training, and the percentage of their current practice that is in primary care pediatrics. Three reported receiving sub-specialty training. Of these, one reported neonatology training, though indicated their current practice to be 90% primary care pediatrics. A second respondent had hematology-oncology training, though reported their current practice to be 100% primary care pediatrics. The third person indicating sub-specialty training is not currently practicing, though indicated that their prior work had been 50% child abuse and 50% general pediatrics. We did not exclude these 3 respondents from our analysis because, though they had sub-specialty training, they indicated their practice experience included primary care pediatrics. When asked to estimate the number of patients in their current practice with an autoimmune disease, the responses ranged from 4 providers who stated they were unsure and did not provide an estimated number, to one provider who stated they follow 100 patients in their practice with an autoimmune disease.

### Section II: general knowledge base

The group was queried regarding the specifics of their training and the adequacy and scope of their pediatric rheumatology instruction. This was accomplished through the following series of questions. Responses were on a 5 point scale, with 1 indicating “strongly agree”, 5 indicating “strongly disagree”.

**Figure 1:**
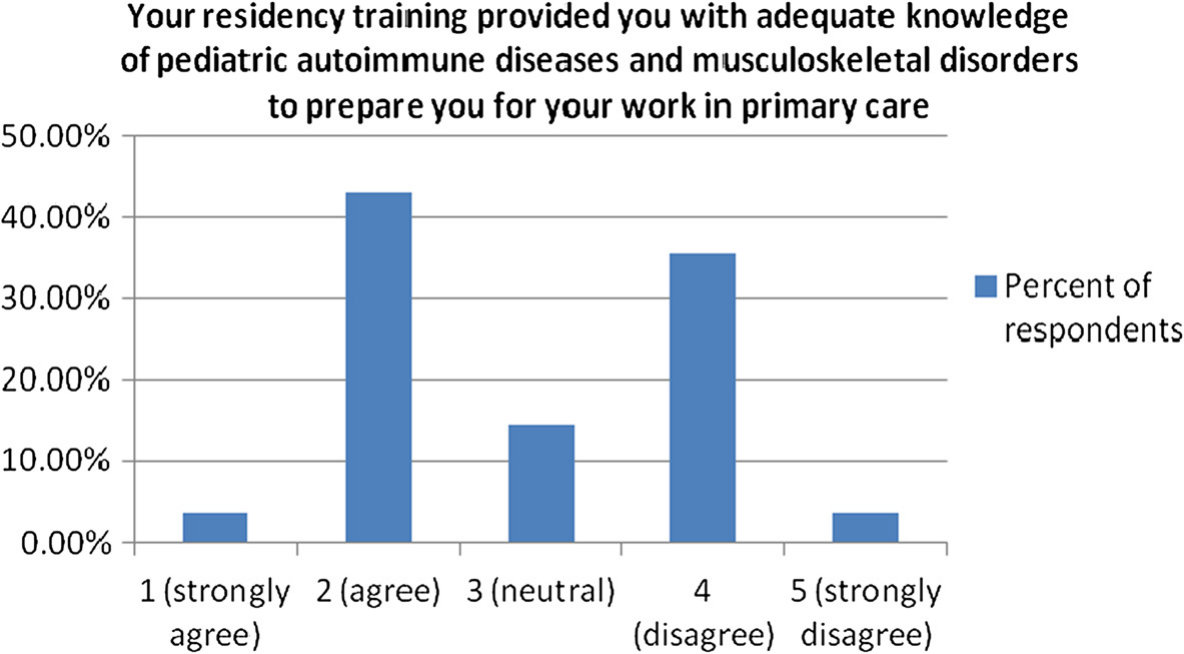
Physician assessment of the adequacy of their residency training in pediatric autoimmune diseases and musculoskeletal disorders.

To the statement “Your residency training provided you with adequate knowledge of pediatric autoimmune diseases and musculoskeletal disorders to prepare you for your work in primary care” 46.5% either agreed or strongly agreed, while 39.3% disagreed or strongly disagreed (Figure 1).

**Figure 2:**
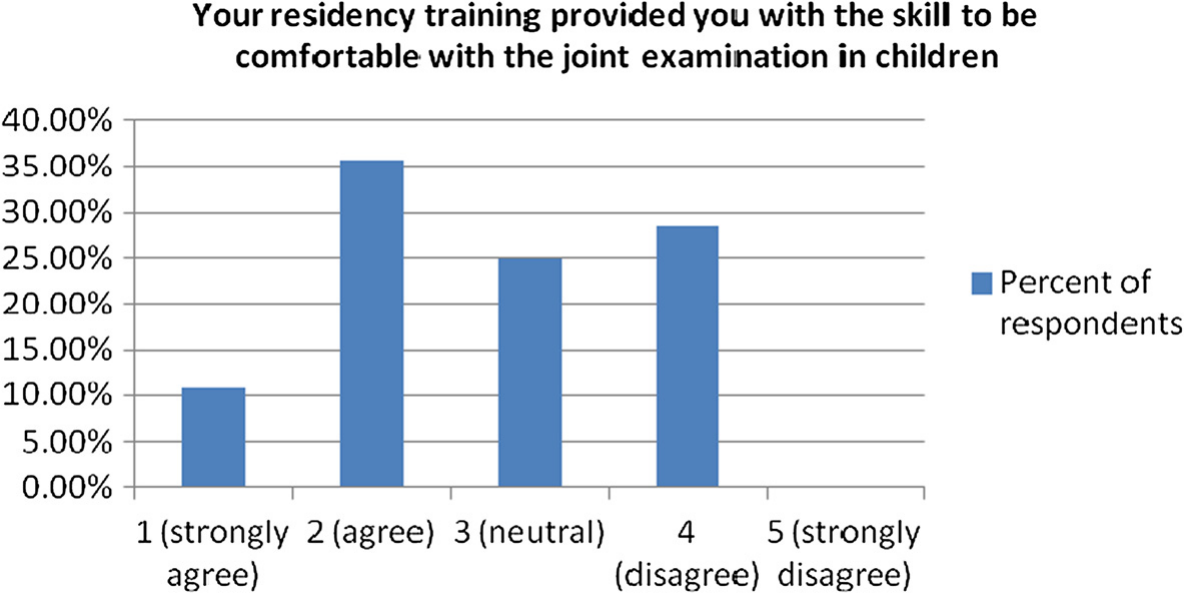
Physician assessment of the adequacy of their residency training in pediatric joint examination.

To the statement “Your residency training provided you with the skill to be comfortable with the joint examination in children” 46.4% either agreed or strongly agreed, while 28.6% disagreed, no respondent strongly disagreed (Figure 2).

**Figure 3:**
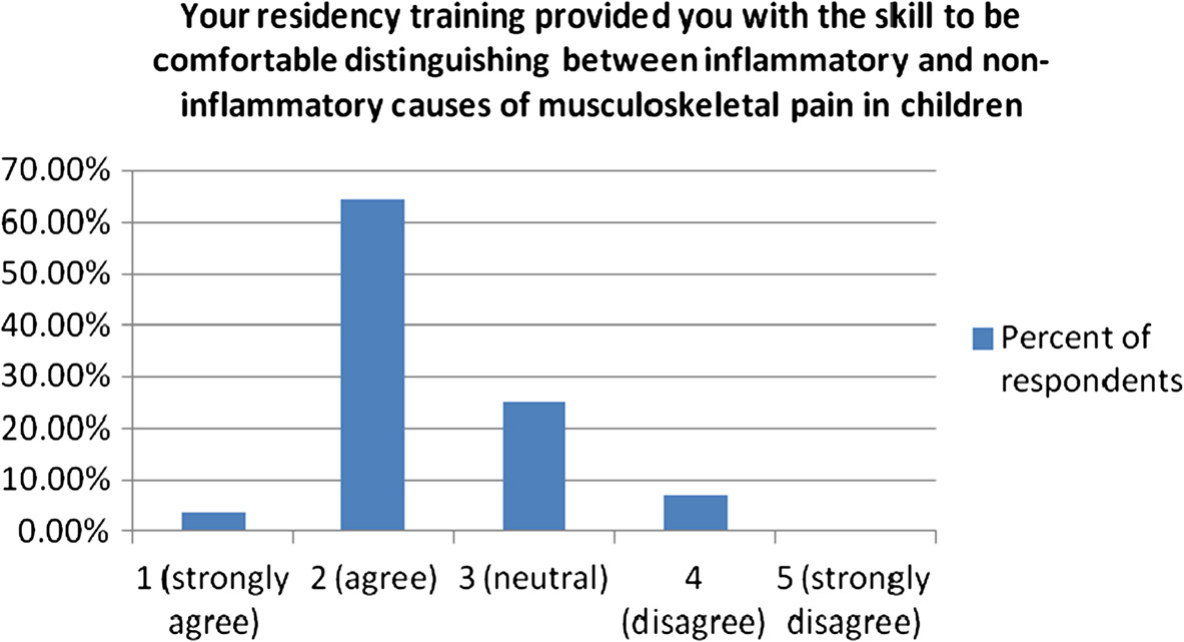
Physician assessment of the adequacy of their residency training in distinguishing between inflammatory and non-inflammatory causes of musculoskeletal pain in children.

To the statement “Your residency training provided you with the skill to be comfortable distinguishing between inflammatory and non-inflammatory causes of musculoskeletal pain in children” 67.9% either agreed or strongly agreed while just 7.1% disagreed, no respondent strongly disagreed (Figure 3).

**Figure 4:**
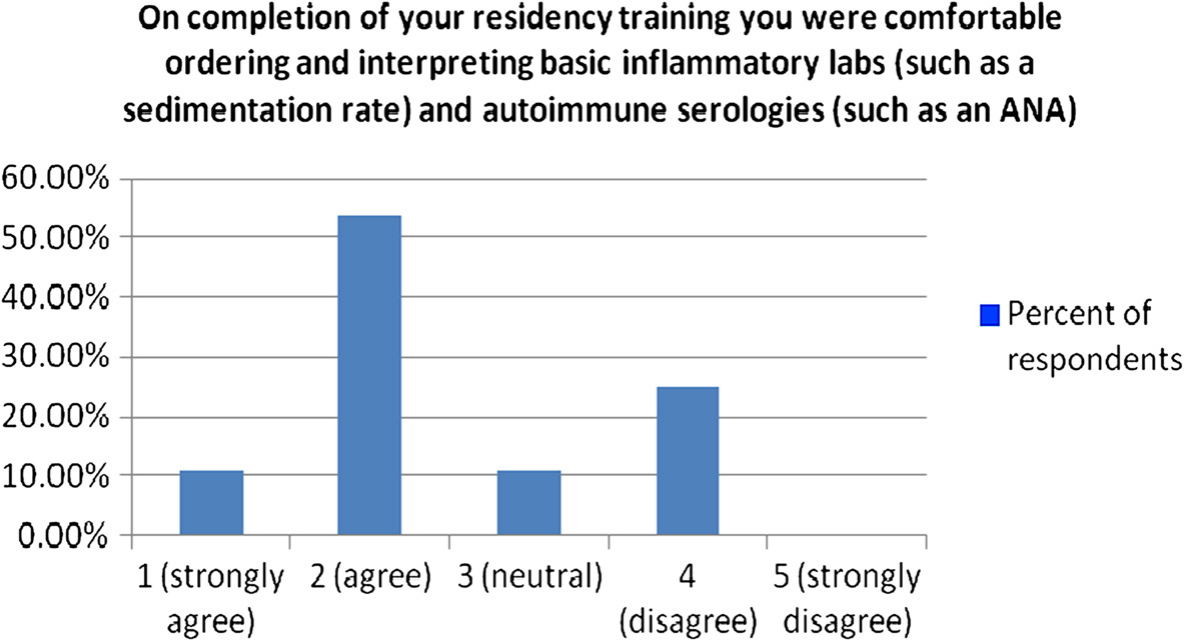
Physician self-assessment of their ability on completion of residency training to order and interpret basic inflammatory labs and autoimmune serologies.

To the statement “On completion of your residency training you were comfortable ordering and interpreting basic inflammatory labs (such as a sedimentation rate) and autoimmune serologies (such as an ANA)” 64.3% either agreed or strongly agreed compared to 25% who disagreed, no respondents strongly disagreed (Figure 4).

**Figure 5:**
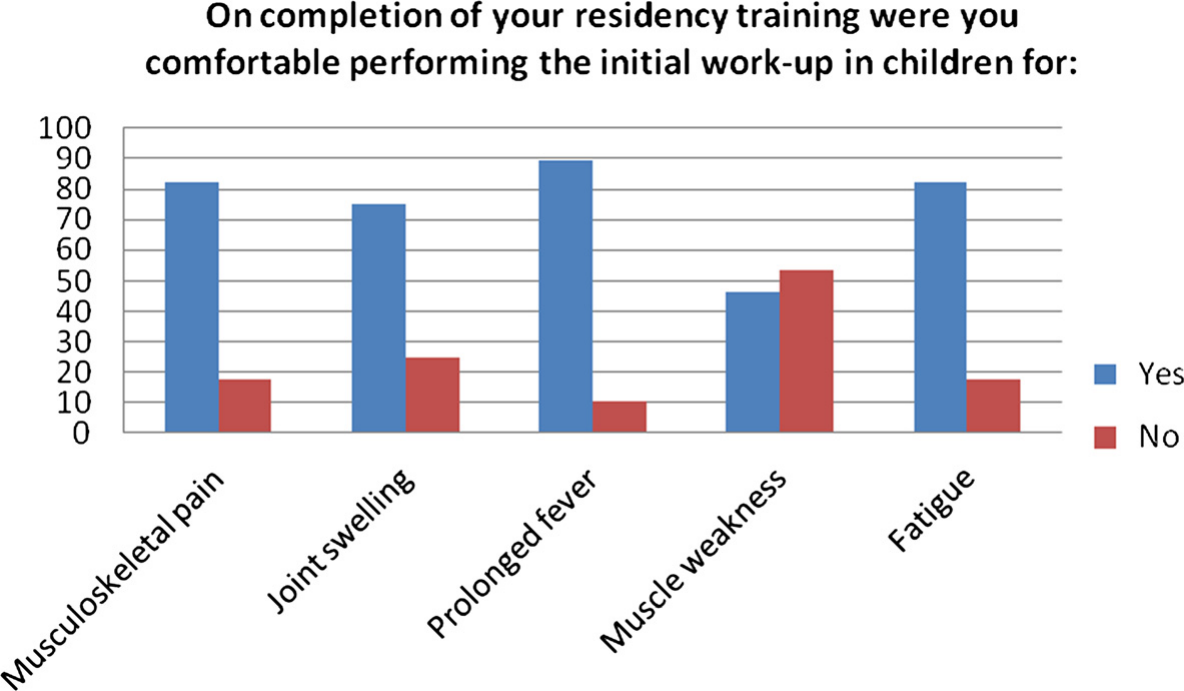
Physician self-assessment of their ability on completion of residency training to perform the initial work-up of presenting complaints referable to rheumatologic diseases in children.

The respondents were then asked to respond Yes or No to the statement “On completion of your residency training were you comfortable performing the initial work-up in children for:” followed by a list of complaints including musculoskeletal pain, joint swelling, prolonged fever, muscle weakness and fatigue. The significant majority of respondents reported leaving residency confident with the initial work-up for all of these complaints with the exception of muscle weakness. Results are shown in Figure 5.

### Section III: knowledge of specific rheumatologic diseases

**Figure 6:**
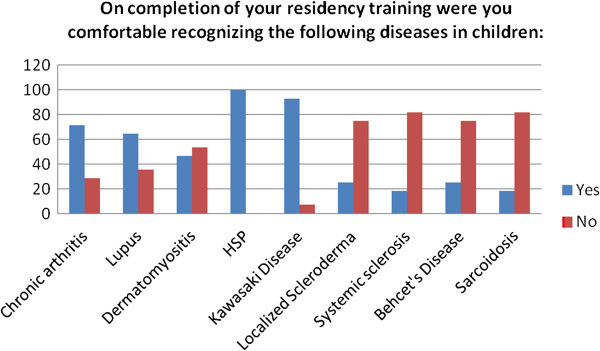
Physician self-assessment of their ability on completion of residency training to recognize specific autoimmune diseases in children.

In this section of the survey, the respondents were asked to respond Yes or No to the statement “On completion of your residency training were you comfortable recognizing the following diseases in children:” followed by a list of 9 rheumatologic diseases. The majority reported leaving residency able to recognize chronic arthritis, systemic lupus erythematosus, Henoch-Schonlein purpura (HSP) and Kawasaki disease, while less than half of the respondents left residency confident in their ability to recognize juvenile dermatomyositis, localized scleroderma, systemic sclerosis (systemic scleroderma), Behcet’s Disease and sarcoidosis. Results are shown in Figure 6.

### Section IV: ability to co-manage patients with their rheumatologist

In this section of the survey we queried the group about co-management of patients with their rheumatologist. We asked their level of agreement with the following statement: “If their autoimmune disease is managed by a rheumatologist, you are comfortable providing primary care, including sick visits, to children with rheumatologic disease”. On the same 5 point scale as used in section II of the survey, 39.3% strongly agreed, 53.6% agreed, 7.1% were neutral, no respondents disagreed or strongly disagreed. In a follow-up question requesting a narrative response, we asked if the respondent is not comfortable with co-management, what factors make this difficult. Of the three responses we received to this question, one pediatrician expressed that “I could use a better understanding of the degree of immunosuppression associated with various drugs/doses”. Another responded with the statement “vaccines are a concern”. The third response was “often lack of communication about where patient is in therapy (ie maintenance, flare, etc.)”.

**Figure 7:**
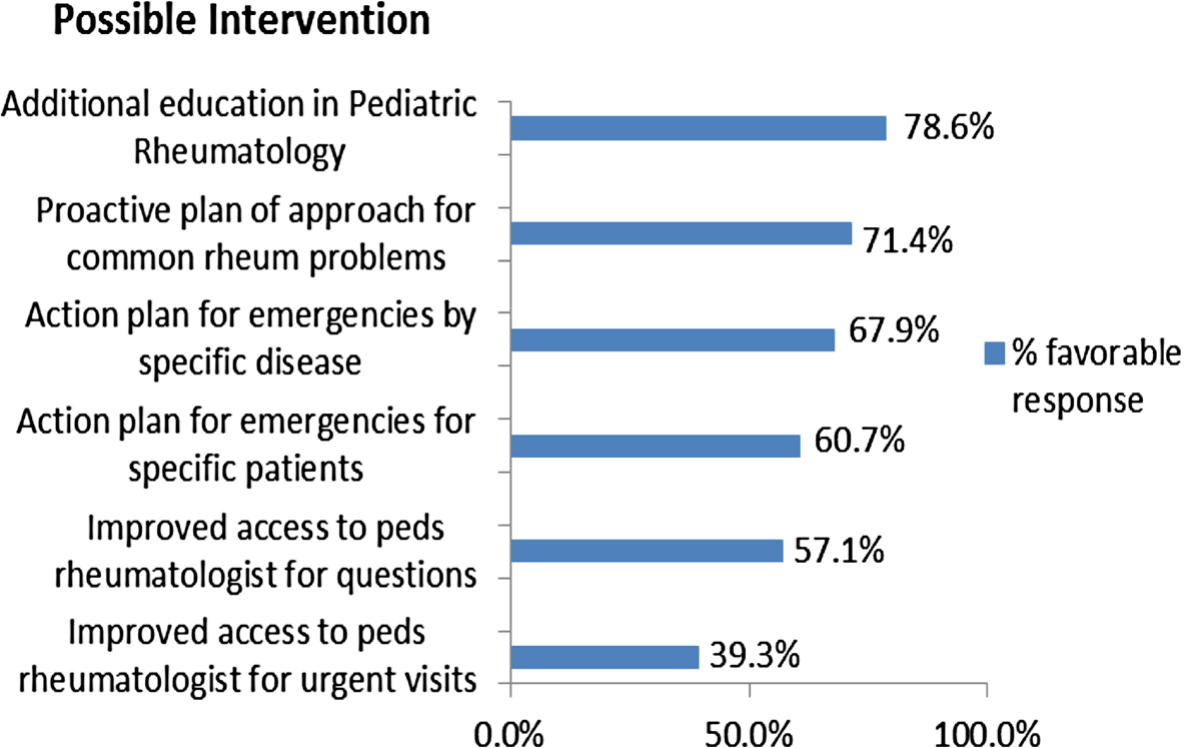
Physician perceptions of the potential utility of interventions to improve their ability to co-manage patients with the rheumatologist.

In an attempt to identify how best to assist the practitioner, the following question was asked: “For your patients with chronic autoimmune diseases, what would improve your ability to co-manage these children with their rheumatologist?” The vast majority of practitioners who answered our survey felt that while having improved access to a pediatric rheumatologist would be a definite benefit, additional education in pediatric rheumatology would be the greatest benefit. Having proactive action plans for common rheumatologic problems as well as for rheumatologic emergencies was also thought to be of potential benefit by the majority of the group (Figure 7).

## Discussion

To our knowledge, ours is the first report of the employment of a needs assessment survey in the development of an educational intervention for primary care physicians in pediatric rheumatology. The major limitation of this study is the low response rate. As this is a common problem with surveys, we took steps during study design to maximize response. These steps included the targeted distrubution of the survey to those graduates who work in primary care and maintain active contact the alumni association, as well as the inclusion of an introductory message of support for the project from the Associate Dean for Alumni Affairs who is known to the graduates. Even with these efforts, our response rate was just 18.4%. It is difficult to know with certainty the reasons behind this low response. It is possible that this represents a selection bias, and those 28 who did repond are actually those physicians who are relatively more aware of and/or knowledgeable about rheumatologic disease than those who did not respond. The fact that 81.6% did not respond may underscore a lack of awareness of rheumatologic disease in primary care practice. The greatest educational need may lie with those physicians who did not respond to our survey.

Another limitation of our study is the fact that this was conducted among residents of a single pediatric residency program. Exposure to pediatric rheumatology varies greatly between residency programs, and the results obtained from Vanderbilt residency graduates may differ from graduates of other programs, thereby limiting the generalizability of our results. From a demographics standpoint, the majority of our repondents practice in a single state (Tennessee), which may further call into question the generalizabilty of our study. The median distance of our respondants’ practice from a pediatric rheumatologist, however, was 50 miles, which is consistent with distances reported nationally [2,8], and therefore suggests that the issue of limited access to pediatric rheumatologic care may be reflected in our data.

Responses to our survey suggest there to be a discrepancy between the physicians’ general assessment of their residency training and their ability to recognize specific diseases. Of our respondents, 46.5% agreed or strongly agreed that they left residency with adequate knowledge of pediatric autoimmune diseases and musculoskeletal disorders for their work in primary care. When queried regarding their ability to perform the initial work-up of a child with muscle weakness, however, 53.6% were uncomfortable with that evaluation, with the same percentage stating they were not confident in their ability to recognize juvenile dermatomyositis. Lupus seemed to be somewhat more familiar to the group, though 35.7% felt they might miss lupus in a child. In addition, 75% were concerned they would miss localized scleroderma, 82% were not confident in recognizing systemic sclerosis, 75% were not comfortable recognizing Behcet’s Disease and 82% were not be comfortable recognizing sarcoidosis.

In contrast, 71.4% of respondents reported confidence in their ability to recognize chronic arthritis. There is the suggestion from these responses that the rarer, though potentially more systemically serious conditions, may be more likely to be missed than chronic arthritis. In looking further at responses related to arthritis, however, there are internal inconsistencies. The high percentage of respondents confident in their ability to recognize chronic arthritis is relatively consistent with the 67.9% who agreed or strongly agreed that they left residency able to distinguish between inflammatory and non-inflammatory causes of musculoskeletal pain in children. This seems inconsistent, however, with the fact that only 46.4% agreed or strongly agreed that they left residency comfortable with the joint exam in a child. Given the centrality of the physical exam in the diagnosis of chronic arthritis, it is difficult to reconcile these responses. In our study 64.3% of respondents agreed or strongly agreed that they left residency comfortable with ordering and interpreting basic inflammatory labs and autoimmune serologies. As has been discussed in other reports [9,10], it may be that our results suggest an inappropriate reliance on laboratory tests in evaluation of joint pain in children by primary care providers.

Looking nationally at available data in the U.S. regarding primary care physicians and juvenile arthritis, a survey of 342 primary care pediatricians and 272 family practice physicians found that 46% of pediatricians and 61% of family practice physicians reported only referring children to confirm a diagnosis of juvenile rheumatoid arthritis and for initial treatment recommendations; these children were not necessarily referred to pediatric rheumatologists [4]. In that same survey, 18% of pediatricians and 12% of family practice physicians reponding to questions regarding self-reported knowledge felt they had adequate training to diagnose/manage juvenile rheumatoid arthritis, while only 10% of pediatricians and 4% of family practice physicians reported being up to date on the latest advances in treatment of juvenile rheumatoid arthritis. This fruther underscores the critical need for better education at the medical school and residency level for physicians who will be caring for children. The need for improved education of trainees has been identifed by educators working in medical systems outside the U.S. as well [11-14].

In his discussion of challenges facing pediatric rheumatology in the arena of resident education, Henrickson discusses what he terms a possible “perceived irrelevance” of pediatric rheumatology among residents [9]. In making this observation, Henrickson cites the low percentage of residents who choose a pediatric rheumatology elective in programs when such an elective is available, estimated to be ≤25% by residency program directors [7]. In a survey of 685 general pediatricians between 1 and 5 years out of residency training, it was reported that 74% responded that they “never or rarely” saw children for whom rheumatologic care was required [15]. This seems difficult to reconcile with the fact that musculoskeletal complaints are one of the most prevalent problems in the pediatric population in the U.S. and abroad [16-18]. In addition, the number of ambulatory visits (office visits and emergency department encounters) in the U.S. for children with a diagnosis of arthritis or other rheumatologic disease for the years 2001–2004 were estimated to be 827,000 [19].

In light of this, perhaps the apparent discrepancy in our survey between the reported adequacy of general knowledge compared to the self-reported inability to recognize many autoimmune diseases reflects the perception, of at least some, that an inability to recognize some of these diseases is reasonable for work in primary care. In our survey, one pediatrician reported having 100 children with an autoimmune disease in their practice, while 4 respondents stated they were not sure enough of the number to provide an estimate. While this may reflect true differences between practices, it also raises the question of whether there exists a wide range of level of awareness of rheumatologic disease.

Primary care is a critically important component of health care for children with chronic illnesses. Among respondents to our survey, 92.9% agreed or strongly agreed that they were comfortable providing primary care for children with rheumatologic disease if a rheumatologist managed their autoimmune disease. Our survey results also indicate that primary care physicians have an interest in practical, problem oriented educational resources to assist them in caring for children with rheumatologic diseases. Nearly 80% of respondents felt that additional education in pediatric rheumatology would improve their ability to co-manage children with the rheumatologist. Having action plans for common rheumatologic complaints and potential emergencies were thought to be of potential benefit by a majority of respondents.

## Conclusions

It is estimated that a 75% increase in the number of pediatric rheumatologists is needed to care for the nearly 300,000 children in the U.S. with rheumatic disease [8]. While there are some workforce gains being made, the reality is that providers other than pediatric rheumatologists will be providing at least some care for children with rheumatic disease for the foreseeable future. We will utilize our survey results to develop curriculum that is self-directed and learner centered to have the highest impact in assisting primary care providers in caring for children with rheumatologic disease. An on-line survey will determine which educational offerings on the site are most effective. As a corollary, we plan to evaluate for possible differences in needs between rural and urban areas. These efforts are undertaken with the ultimate goal of avoiding delayed or missed diagnoses of treatable autoimmune diseases in children, thereby minimizing poor outcomes through timely and appropriate care.

## Competing interests

The authors declare that they have no competing interests.

## Authors’ contributions

ALW and ZLH both participated in study design, data analysis and manuscript preparation. Both authors read and approved the final manuscript.
